# In Vitro Susceptibility to Ceftazidime/Avibactam and Comparators in Clinical Isolates of Enterobacterales from Five Latin American Countries

**DOI:** 10.3390/antibiotics9020062

**Published:** 2020-02-05

**Authors:** Tobias Manuel Appel, María Fernanda Mojica, Elsa De La Cadena, Christian José Pallares, Marcela A. Radice, Paulo Castañeda-Méndez, Diego A. Jaime-Villalón, Ana C. Gales, José M. Munita, María Virginia Villegas

**Affiliations:** 1 Grupo de Resistencia Antimicrobiana y Epidemiología Hospitalaria (RAEH), Universidad El Bosque, Bogotá 110121, Colombia; tobiasm.appel@gmail.com (T.M.A.); Maria.MojicaMedina@va.gov (M.F.M.); ecadenav@unbosque.edu.co (E.D.L.C.); icako@hotmail.com (C.J.P.); 2 Grupo de Resistencia Bacteriana, Centro Internacional de Entrenamiento e Investigaciones Médicas (CIDEIM), Cali 760031, Colombia; 3 Comité de Infecciones y Vigilancia Epidemiológica, Centro Médico Imbanaco, Cali 760043, Colombia; 4 Departamento de Microbiología, Inmunología, Biotecnología y Genética, Cátedra de Microbiología, Universidad de Buenos Aires—CONICET, Buenos Aires C1113AAD, Argentina; mradice@ffyb.uba.ar; 5 Department of Infectious Diseases, Hospital Médica Sur, Ciudad de México 14050, Mexico; drpaulocastanedam@hotmail.com; 6 Department of Infectious Diseases, Hospital San Angel Inn Universidad, Ciudad de México 03330, Mexico; djaime_md@hotmail.com; 7 Department of Internal Medicine, Division of Infectious Diseases, Universidade Federal de São Paulo, São Paulo 04039-032, Brazil; ana.gales@gmail.com; 8 Genomics and Resistant Microbes (GeRM) Group, Millennium Initiative for Collaborative Research On Bacterial Resistance (MICROB-R), Santiago, Región Metropolitana 7650568, Chile; munita.jm@gmail.com

**Keywords:** Antimicrobial activity, Argentina, Brazil, Chile, Colombia, Mexico

## Abstract

*Background*: High rates of resistance to third-generation cephalosporins and carbapenems in Enterobacterales have been reported in Latin America. Ceftazidime/avibactam (CZA) is the combination of a third-generation cephalosporin and a non-β-lactam β-lactamase inhibitor, which has shown activity against isolates producing class A, C and D β-lactamases. Herein, we evaluated the activity of CZA and comparators against clinical isolates of Enterobacterales in Latin America. *Methods*: The activity of CZA and comparators was evaluated against clinical isolates of Enterobacterales from Argentina, Brazil, Chile, Colombia and Mexico that were collected between January 2016 and October 2017. One specific phenotypic subset was evaluated. A carbapenem non-susceptible (CNS) phenotype was defined as any isolate displaying a minimum inhibitory concentration (MIC) ≥1 mg/L for ertapenem. *Results*: CZA was active against 95.8% of all isolates and 77.5% of CNS isolates. Fosfomycin (FOS) and tigecycline (TGC) were the second most active antibiotics with 93.4% of Enterobacterales being susceptible. *Conclusions*: The results of this study underline the potential therapeutic role of CZA in Latin America.

## 1. Introduction

Antimicrobial resistance is a threat to public health. Enterobacterales are some of the most common and pathogenic microorganisms that have acquired resistance to several classes of antimicrobials [1]. Particularly concerning is the resistance to carbapenems since these agents are often considered the last resort antibiotics. In addition, infections caused by carbapenem-resistant enterobacteria are associated with higher costs and mortality rates [2,3]. 

The most frequently found carbapenem resistance mechanism is the production of carbapenemases, among which *Klebsiella pneumoniae* carbapenemases (KPC) are the most widely distributed worldwide and are endemic in several countries of the Latin American region [4]. Ceftazidime/avibactam (CZA) is the combination of a third-generation cephalosporin and a non-β-lactam inhibitor capable of inhibiting several class D, C and A β-lactamases, including the KPC-family enzymes. Several in vitro, in vivo and clinical studies have reported favorable results with CZA against carbapenemase-producing enterobacteria, while being less toxic than other agents commonly used to treat carbapenem-resistant bacteria, such as colistin and aminoglycosides [5,6,7].

Herein, we evaluated the activity of CZA and comparators against 2252 clinical isolates of Enterobacterales from 20 healthcare institutions located in Argentina, Brazil, Chile, Colombia, and Mexico between January 2016 and October 2017.

## 2. Results

The distribution of the 2252 isolates of Enterobacterales per country and species is shown in Table 1. Overall, 95.8% (2158/2252) of the isolates were susceptible to CZA (minimum inhibitory concentration of 90% of isolates (MIC_90_) ≤1 mg/L). The highest susceptibility was observed in *Escherichia coli* (97.9%), followed by *Serratia marcescens* (94.5%), *Klebsiella aerogenes* (93.3%), *Klebsiella pneumoniae* (92.1%) and isolates of the *Enterobacter cloacae* complex with a susceptibility of 92.0% (Table 2). Fosfomycin (FOS) and tigecycline (TGC) were the second most active antibiotics with 93.4% of Enterobacterales susceptible, followed by the carbapenems meropenem (MEM) (88.7%), imipenem (IMI) (87.1%) and ertapenem (ETP) (82.4%).

In all five countries, the susceptibility of Enterobacterales to CZA was similarly high, ranging from 99.1% in Chile (MIC_90_ ≤ 1 mg/L), 98.9% in Mexico (MIC_90_ ≤ 1 mg/L), 97.4% in Argentina (MIC_90_ ≤ 1 mg/L), 96.5% in Brazil (minimum inhibitory concentration of 50% of isolates (MIC_50_) ≤ 1 mg/L, MIC_90_ 2 mg/L) to 94.3% in Colombia (MIC_50_ ≤ 1 mg/L, MIC_90_ 2 mg/L). Comparable results were observed for FOS (92.5%–97.4%) and TGC (81.5%–95.8%). For carbapenem non-susceptible (CNS) Enterobacterales, CZA was active against 77.5% of all tested strains (MIC_50_ 2 mg/L, MIC_90_ ≥ 128 mg/L). The activity of CZA was the highest in CNS isolates from Chile (94.8%, MIC_50_ 2 mg/L, MIC_90_ 8 mg/L), followed by Mexico (93.3%, MIC_50_ ≤ 1 mg/L, MIC_90_ 1 mg/L), Brazil (88.6%, MIC_50_ ≤ 1 mg/L, MIC_90_ 32 mg/L), Argentina (80%, MIC_50_ ≤ 1 mg/L, MIC_90_ 64 mg/L), and Colombia (71.3%, MIC_50_ 2 mg/L, MIC_90_ ≥ 128 mg/L) (Table 1). 

For all species of Enterobacterales, regardless of their susceptibility profile, CZA was the compound with the highest activity when compared with other β-lactam agents. For isolates of *E. coli* and *E. cloacae* complex, CZA was superior to all other antimicrobials tested. In the case of *K. pneumoniae* and *K. aerogenes,* the activity of FOS was slightly superior to CZA, whereas for *S. marcescens* both antimicrobials showed a susceptibility of 94.5%. 

From the 2252 isolates tested, 396 (17.6%) were found to be CNS; of note, 46.2% were identified as *K. pneumoniae*. CZA was active against 77.5% of the CNS isolates (MIC_50_ 2 mg/L, MIC_90_ ≥ 128 mg/L), with the highest activity against *S. marcescens* (81.5%), while the lowest susceptibility was observed for *K. pneumoniae* (74.3%). For this group, the activity of CZA was superior to all β-lactams and superior or equal to that of FOS for isolates of *E. cloacae* complex, *K. aerogenes* and *S. marcescens*.

## 3. Discussion

This study showed that 95.8% of clinical isolates of Enterobacterales from five Latin American countries, collected between January 2016 and October 2017, were susceptible to CZA (MIC_90_ ≤ 1 mg/L). The susceptibility to CZA between species ranged from 97.9% for *E. coli* to 92.0% for isolates of *E. cloacae* complex. Furthermore, 77.5% of CNS isolates remained susceptible to CZA. These results underline the potential therapeutic role of CZA for patients infected with KPC-producing and other carbapenemase-producing enterobacteria, which are prevalent in the Latin American region [4,7]. 

Although the present study might be limited by the small number of isolates from Mexico and Brazil and the fact that they are from a single center in Argentina, Brazil and Mexico, our results are similar to most reports described previously by other authors. In a study by Flamm et al. [8], CZA was evaluated against 130 clinical urinary isolates of Enterobacterales collected in 2011 from Argentina, Brazil, Chile, Colombia, Mexico, Panama and Venezuela, finding a MIC_90_ of 0.25 mg/L. Of the evaluated strains, 0.8% were resistant to MEM. Similarly, Karlowsky et al. [9] evaluated the activity of CZA and comparators against clinical isolates of Enterobacterales and *P. aeruginosa* collected between 2012 and 2015 from six Latin American countries (Argentina, Brazil, Chile, Colombia, Mexico and Venezuela). In this study, CZA was active against 99.7% of 7665 Enterobacterales, which is similar to our findings. Furthermore, 5.1% of all isolates were carbapenem (MEM) non-susceptible. In the MEM non-susceptible subgroup, the authors observed that CZA was active against 95.4% of isolates, which is significantly higher compared to our observations. 

The differences in CZA susceptibility of the non-susceptible subgroups could be explained by the different hospitals and geographical areas included in the study, as well as the changes in the epidemiology of resistance mechanisms between the study periods. For example, in the case of Brazil, susceptibility rates to CZA in this study were inferior to those observed previously against *K. pneumoniae* isolates in a surveillance study by Rossi et al. (100% susceptible) [10]. An increase in class B β-lactamases (which were detected in 0.2% of all Enterobacterales by Karlowsky et al.) or the emergence of different mechanisms of resistance to CZA in class A β-lactamase-producing *K. pneumoniae* as reported in the literature could explain this difference [11,12]. 

## 4. Materials and Methods

Isolates were collected in each of the participating institutions between January 2016 and October 2017. Upon reception, species confirmation was performed using matrix-assisted laser desorption/ionization time-of-flight mass spectrometry (Biomeriéux, Marcy-l’Étoile, France). Susceptibility testing was performed in the laboratory of the research group Resistencia Antimicrobiana y Epidemiología Hospitalaria (RAEH), Universidad El Bosque, Bogotá, Colombia. Minimum inhibitory concentrations (MICs) were determined by broth microdilution using customized Sensititre plates (TREK Diagnostic Systems, East Grinstead, West Sussex, UK), with *E. coli* ATCC 25922 as quality control, following Clinical and Laboratory Standards Institute (CLSI) guidelines [13]. Antibiotics evaluated included: ceftazidime/avibactam (CZA; 1/4–128/4 mg/L), ceftazidime (CAZ; 2–32 mg/L), cefepime (FEP; 2–64 mg/L), piperacillin/tazobactam (TZP; 2/4–128/4 mg/L), ertapenem (ETP; 0.25–32 mg/L), imipenem (IMP; 0.25–128 mg/L), meropenem (MEM; 0.25–128 mg/L), tigecycline (TGC; 0.25–8 mg/L) and fosfomycin (FOS; 8–128 mg/L). With the exception of FOS and TGC, results were interpreted according to the CLSI 2018 breakpoints [14]. FOS breakpoints for Enterobacterales were extrapolated from the *E. coli* breakpoint by CLSI (FOS non-susceptible MIC ≥128 mg/L). United States Food and Drug Administration product package insert criteria were used as breakpoints for TGC (susceptible: ≤2 mg/L; intermediate: 4 mg/L; resistant: ≥8 mg/L) [15]. The specific phenotypic subset defined as a carbapenem non-susceptible (CNS) phenotype included isolates displaying a MIC ≥1 mg/L for ETP.

## 5. Conclusions

We report excellent activity of CZA against diverse Enterobacterales collected in Latin America. The lower rates of CZA susceptibility among CNS isolates in our study highlights the importance of active surveillance programs in order to follow the evolution of resistance mechanisms against the antibiotic armamentarium, including newly introduced antimicrobial agents. 

## Figures and Tables

**Table 1:** Susceptibility of Enterobacterales to ceftazidime/avibactam and comparators by country.

Microorganism	Number of Isolates	Percentage of Susceptibility								
		CZA	CAZ	FEP	TZP	ETP	IMI	MEM	TGC	FOS
**Argentina**	233									
*E. coli*	160	97.5	53.8	91.3	60	95.6	96.3	96.9	98.1	98.1
CNS	7	57.1	0	14.3	14.3	-	14.3	28.6	28.6	57.1
*K. pneumoniae*	65	98.5	52.3	61.5	49.2	81.5	87.7	89.2	93.8	96.9
CNS	12	100	8.3	8.3	8.3	-	33.3	41.7	75	91.7
*E. cloacae* complex	4	100	75	75	75	75	75	75	75	75
CNS	0									
*S. marcescens*	4	75	75	75	75	75	75	75	75	75
CNS	1	0	0	0	0	-	0	0	0	0
**Brazil**	85									
*E. coli*	20	95	65	65	80	70	75	75	90	100
CNS	6	83.3	14.3	14.3	42.9	-	14.3	14.3	57.1	85.7
*K. pneumoniae*	23	87	4.3	8.7	13	21.7	17.4	21.7	73.9	95.7
CNS	18	83.3	0	0	0	-	0	0	66.7	94.4
*E. cloacae* complex	24	100	25	29.2	58.3	62.5	83.3	87.5	79.2	79.2
CNS	9	100	0	11.1	44.4	-	66.7	55.6	66.7	77.8
*S. marcescens*	18	100	100	61.1	66.7	83.3	88.9	83.3	88.9	83.3
CNS	2	100	0	0	0	-	0	0	50	100
**Chile**	443									
*E. coli*	347	99.1	70.3	76.7	91.1	88.8	94.2	96.5	94.5	94.8
CNS	39	94.9	23.1	25.6	51.3	-	53.8	69.2	59	92.3
*K. pneumoniae*	66	98.5	43.9	51.5	60.6	78.8	90.9	83.3	93.9	90.9
CNS	14	92.9	0	0	14.3	-	57.1	21.4	92.9	71.4
*E. cloacae* complex	21	100	81	100	90.5	90.5	100	100	95.2	85.7
CNS	2	100	100	100	50	-	100	100	100	100
*S. marcescens*	9	100	66.7	66.7	77.8	66.7	100	88.9	100	100
CNS	3	100	33.3	33.3	33.3	-	100	66,7	100	100
**Colombia**	1396									
*E. coli*	813	97.3	79.3	81.8	91.4	90.7	94.7	95.1	96.1	94.1
CNS	76	72.4	0	0	0	-	44.7	47.4	64.5	76.3
*K. pneumoniae*	441	90.2	52.4	56.7	61.7	68.9	74.1	76.2	91.2	91.6
CNS	137	68.6	0	0	0	-	18.2	23.4	73	80.3
*E. cloacae* complex	82	87.8	47.6	47.6	54.9	58.5	78	78	90.2	80.5
CNS	34	73.5	11.8	11.8	20.6	-	29.4	38.2	76.5	70.6
*S. marcescens*	60	93.3	61.7	63.3	63.3	65	66.7	73.3	76.7	93.3
CNS	21	81	4.8	4.8	19	-	19	23.8	52.4	81
**Mexico**	95									
*E. coli*	69	100	34.8	39.1	73.9	87	91.3	97.1	95.7	94.2
CNS	9	100	11.1	0	11.1	-	44.4	77.8	66.7	11.1
*K. pneumoniae*	15	100	66.7	66.7	40	86.7	86.7	86.7	100	100
CNS	2	100	0	0	0	-	50	50	100	100
*E. cloacae* complex	11	90.9	27.3	18.2	9.1	63.6	18.2	72.7	90.9	100
CNS	4	75	0	0	0	-	25	25	100	100

CAZ: ceftazidime; CZA: ceftazidime/avibactam; ETP: ertapenem; FEP: cefepime; FOS: fosfomycin; IMI: imipenem; MEM: meropenem; TGC: tigecycline; TZP: piperacillin/tazobactam

**Table 2:** Susceptibility of Enterobacterales to ceftazidime/avibactam according to minimum inhibitory concentration (MIC) (mg/L) distribution and susceptibility to comparators.

Microorganism	Number of Isolates	Ceftazidime/Avibactam											Susceptibility to Comparators (% Isolates Susceptible)							
		Cumulative Percentage of Isolates at Each MIC (mg/L)																		
		≤1	2	4	8	16	32	64	≥128	MIC_50_	MIC_90_	%S	CAZ	FEP	TZP	ETP	IMI	MEM	TGC	FOS
Enterobacterales	2252	89	93.8	95.2	95.8	96.1	96.9	97.7	100	≤1	2	95.8	64	67.7	79	82.4	87.1	88.7	93.4	93.4
CNS	396	44.7	66.9	74.7	77.5	78.8	83.1	87.6	100	2	≥128	77.5	8.6	12.1	26.8	-	31.6	35.9	68.9	81.3
*E. coli*	1409	93.4	96.9	97.6	97.9	98.2	98.5	99	100	≤1	≤1	97.9	71.8	75.7	90.3	90.3	91.8	95.5	95.6	94.8
CNS	137	43.8	70.1	77.4	80.3	81.8	84.7	89.1	100	2	≥128	80.3	16.8	21.9	46.7	-	44.5	53.3	61.3	82.5
*K. pneumoniae*	610	81.8	88.2	91	92.1	92.4	93.9	95.4	100	≤1	4	92.1	50	53.4	59.2	70	75.6	76.6	91.3	92.5
CNS	183	45.4	62.9	71.6	74.3	75.4	80.3	85.2	100	2	≥128	74.3	2.7	4.9	13.7	-	20.8	22.4	74.3	82
*E. cloacae* complex	112	79.5	88.4	90.2	92	92	96.5	97.4	100	≤1	4	92	42.9	46.4	51.8	63.4	71.4	79.5	79.5	79.5
CNS	41	46.3	70.7	75.6	80.5	80.5	90.3	92.7	100	2	32	80.5	7.3	14.6	19.5	-	39	43.9	80.5	73.2
*K. aerogenes*	30	86.7	90	93.3	93.3	96.6	96.6	100	100	≤1	2	93.3	66.7	70	83.3	73.3	83.3	83.3	90	96.7
CNS	8	50	62.5	75	75	87.5	87.5	100	100	≤1	64	75	12.5	12.5	50	-	37.5	37.5	50	75
*S. marcescens*	91	81.3	92.3	94.5	94.5	94.5	94.5	95.6	100	≤1	2	94.5	62.6	64.8	69.2	70.3	73.6	78	80.2	94.5
CNS	27	40.7	74	81.4	81.4	81.4	81.4	85.1	100	2	≥128	81.5	7.4	7.4	18.5	-	25.9	25.9	55.6	81.5

CAZ: ceftazidime; CZA: ceftazidime/avibactam; ETP: ertapenem; FEP: cefepime; FOS: fosfomycin; IMI: imipenem; MEM: meropenem; MIC_50_: minimum inhibitory concentration of 50% of isolates; MIC_90_: minimum inhibitory concentration of 90% of isolates; TGC: tigecycline; TZP: piperacillin/tazobactam; %S: isolates susceptible.

## Abbreviations

CAZ	Ceftazidime
CNS	Carbapenem non-susceptible
CZA	Ceftazidime/avibactam
ETP	Ertapenem
FEP	Cefepime
FOS	Fosfomycin
IMI	Imipenem
MEM	Meropenem
MIC	Minimum inhibitory concentration
TGC	Tigecycline
TZP	Piperacillin/tazobactam
